# Prospective, monocentric, observational study of the long-term effectiveness of omalizumab in chronic urticaria 

**DOI:** 10.5414/ALX02376E

**Published:** 2023-01-03

**Authors:** Stephan Traidl, Bettina Wedi

**Affiliations:** Department of Dermatology and Allergy, Hannover Medical School, Hannover, Germany

**Keywords:** omalizumab, chronic urticaria, effectiveness, real-word data

## Abstract

Omalizumab, a monoclonal antibody targeting IgE, has been approved in 2014 for the treatment of H1 antihistamine-refractory chronic urticaria. Data on long-term effectiveness and predictive factors for treatment response are currently limited. In this monocentric, prospective, observational study, 112 patients with chronic spontaneous urticaria (CSU) and 32 patients with chronic inducible urticaria (CIndU) were included. In addition to a rapid response, omalizumab also showed good effectiveness on both forms of chronic urticaria over 2 years. Low total IgE and elevated *Yersinia* IgA were identified as potential predictive markers for slower treatment responses in CSU. In conclusion, the present study highlights the efficacy of omalizumab for the treatment of chronic urticaria. With emerging new therapeutic options for chronic urticaria, further genetic as well as molecular markers are needed to establish patient-specific therapy selection.

## Introduction 

Chronic urticaria is manifested by wheals and/or angioedema that recur for at least 6 weeks. This occurs either spontaneously or as a result of external stimuli, e.g., cold or pressure. Accordingly, two subtypes are differentiated: chronic spontaneous urticaria (CSU) and chronic inducible urticaria (CIndU) [1]. With regard to the pathophysiology, there is a release of mediators, particularly histamine, from mast cells and basophil granulocytes that induce marked pruritus. This leads to a pronounced impairment of quality of life, causing significant direct as well as indirect costs [2]. Based on the pathomechanism, two types are distinguished in CSU: type I and IIb [3]. While type I is characterized by, among others, anti-TPO IgE antibodies, type IIb shows IgG-mediated autoimmune processes at the high-affinity IgE receptor (FcεRI). 

Due to the identified relevance of IgE and FcεRI, studies investigating the efficacy of omalizumab, a monoclonal anti-IgE antibody, followed. The phase 3 study showed a significant reduction of itch after 12 weeks compared to placebo [4]. Omalizumab subsequently received approval for CSU in 2014 (and for chronic rhinosinusitis with nasal polyposis in 2020). Although omalizumab is not approved for CIndU, it showed good efficacy in most patients [5, 6]. 

To date, prospective data from real-world care are limited. Ertas et al. demonstrated that initial serum total IgE can be used as a predictive marker for response [7]. In the present prospective study, the analysis of the change in urticaria control as well as quality of life collected over up to 2 years of omalizumab therapy was performed. 

## Materials and methods 

The present study is a monocentric, prospective, observational study. The study population was formed from all adult patients who completed the preliminary examinations and received at least one injection of omalizumab between January 2014 and January 2020 in the Department of Dermatology, Allergology and Venereology of the Hannover Medical School (MHH). Before starting therapy, a variety of clinical as well as laboratory parameters were collected and evaluated in the analyses. Clinical parameters were gender, smoking status, type of urticaria, age at onset, and age at start of therapy. Laboratory chemistry included *Helicobacter* stool antigen test, *Yersinia* serology (IgA, IgG, IgM, immunoblot), differential blood count, CRP, TSH, thyroid autoantibodies (MAK, TAK, TRAK), total IgE, and Sx1 (inhalation screen). Furthermore, an autologous serum test was performed in a large proportion of patients [8]. At each visit, the urticaria control test (UCT) and the Quality of Life Questionnaire for Chronic Urticaria (CU-Q2oL) were completed by the patients and noted in a list. The list was digitized at regular intervals. The laboratory chemistry data were transferred from the hospital management system to a digital table twice and subsequently compared to exclude false transmissions. The 12-month time point was considered with a 1-month tolerance, i.e., 11 – 13 months after the start of omalizumab therapy, and the 24-month value with a 2-month tolerance. 

### Statistics 

General characteristics were analyzed using descriptive statistics and statistically compared according to scale level using the Mann-Whitney U test and the χ^2^-test. For comparison of changes from baseline, ANOVA was performed followed by Bonferroni-corrected post-hoc analysis. For consideration of factors influencing response, “responder” was defined as improvement in UCT score by at least 3 points at 1 month. This corresponds to the described minimal clinical difference (MCD) for UCT [9]. Binary logistic regression was performed in each case to determine the odds ratio with 95% confidence interval. Multivariate analysis was also performed with all significant influencing factors followed by backward selection. Data were compiled using Excel (version 16.64, Microsoft, Redmond, WA, USA). Analysis was performed using SPSS (version 27, IBM Corporation, Armonk, NY, USA) and R (version 4.1.2, RStudio, PBC, Boston, MA, USA). Correlations were calculated using the function “cor” (pairwise.complete) of the package “stats”, version 3.6.2, and the significance of the correlations was determined using the function corr.mtest of the package “corrplot_0.92”. A correction for multiple testing was made using the Benjamini-Hochberg method. The α-error was set to 0.05 before correction. 

## Results 

From 2014 to 2019, therapy with omalizumab was documented in 161 patients. 17 patients were excluded for further analysis because (1) the diagnosis included only histamine-mediated angioedema (n = 7), (2) initiation did not occur at MHH (n = 4), (3) the patient initially received omalizumab as part of a study (n = 4), and (4) the diagnosis changed during the course (n = 2). Of the 144 patients, 112 (77.8%) experienced CSU exclusively and in 53.6% with concomitant angioedema (Table 1). In 22.2% of the omalizumab patients, at least one form of CIndU was present, with 46.9% also suffering from concomitant CSU. CIndU patients developed CSU on average slightly earlier but received omalizumab therapy significantly later (44.3 ± 74.3 vs. 50.6 ± 51.4 months), with a wide range in both groups from a few weeks to > 10 years. Regarding smoking, there was a trend for CIndU patients to report smoking slightly more frequently. 

**Figure 1:**
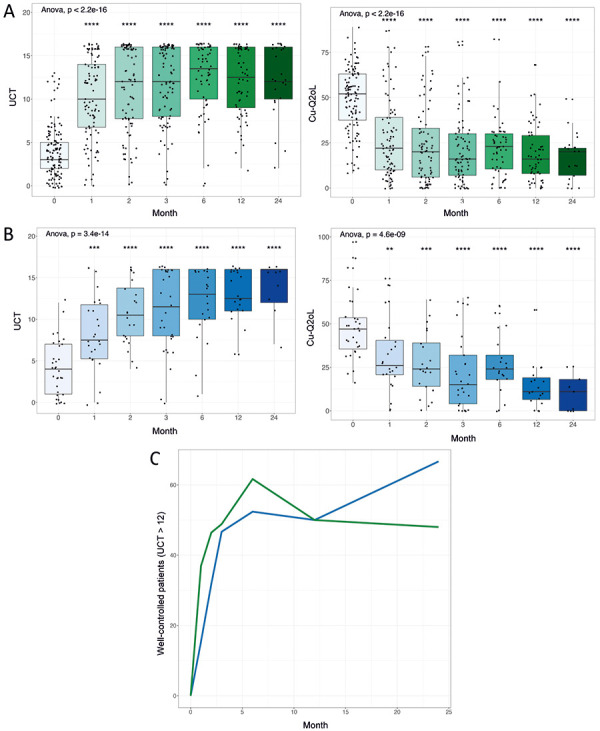
Response of patients with chronic spontaneous urticaria (CSU) and chronic inducible urticaria (CIndU) over a total of 24 months based on UCT and CU-Q2oL. A: boxplot for CSU, left: UCT, right: CU-Q2oL. B: Figures for CIndU, left: UCT, right: CU-Q2oL. C: analysis of the proportion of well-controlled patients, defined as UCT > 12 points.

To analyze the effectiveness of omalizumab for the treatment of chronic urticaria, the change in UCT as well as CU-Q2oL after 1, 2, 3, 6, 12, and 24 months were considered first. There was a significant improvement in urticaria control as well as quality of life already after 1 month (Figure 1A). A further improvement of the values was observed over the following months. There was also a significant reduction in the frequency of H1 antihistamines taken after 1 month (data not shown). 

With regard to the CIndU patients, a significant improvement in urticaria control and quality of life was also observed after 1 month (Figure 1B). Comparatively, the proportion of patients with good control of urticaria, defined as UCT > 12 points, showed a significantly higher proportion of patients with well-controlled disease in CSU after 1 and after 2 months. In both CSU and CIndU, additional responders continued to emerge after 3 months of omalizumab therapy, and in CIndU, further improvements in urticaria control occurred even after 12 months (Figure 1C). 

Omalizumab therapy was well tolerated by all patients. No relevant adverse events were reported. 

To analyze the extent to which pre-treatment testing and clinical characteristics can predict a rapid response to omalizumab, the CSU study population was divided into responders and non-responders (at least 3 points improvement in UCT after 1 month) (Table 2). This showed that female gender was a positive predictor of rapid response in the present cohort. A positive autologous serum skin test (ASST) was associated with a worse response to omalizumab: improvement by 3 UCT points at 1 month was reduced by 66%. Likewise, low total IgE before initiation of therapy with omalizumab was a negative predictor, with IgE < 25 kU/L in particular reducing the odds ratio of response by a factor of 10 after 1 month. Other clinical or serologic parameters showed no effect on response at 1 month. Multivariate logistic regression yielded that low total IgE was the most important factor (IgE < 25 kU/L, OR 0.13, 95% CI: 0.04 – 0.48) predicting slower response. 

**Figure 2:**
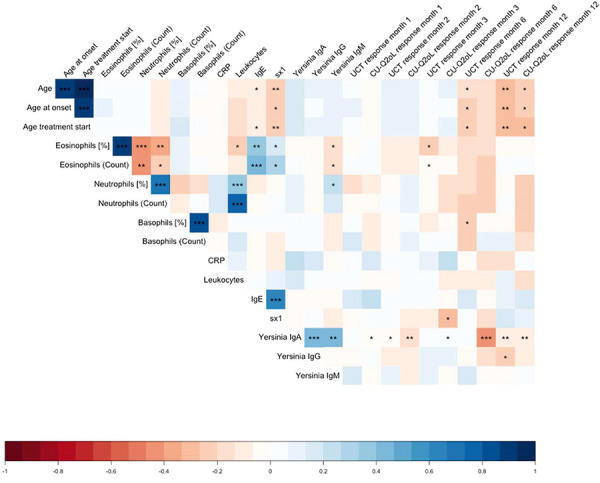
Correlation of different patient characteristics as well as blood values with UCT and CU-Q2oL response at 1, 2, 3, 6 and 12 months. * < 0,05, ** < 0,01, *** < 0,001.

For further analyses regarding the influence of different factors on the improvement of urticaria control over time, a correlation matrix was constructed (Figure 2). In particular, a negative correlation of *Yersinia* IgA with improvement in quality of life at 2, 6, and 12 months was evident, i.e., increased *Yersinia* IgA was associated with reduced improvement in quality of life (as well as control of urticaria at 12 months). Furthermore, long-term response over 12 months correlated negatively with current age and age at initial manifestation. 

## Discussion 

The results of this prospective long-term observational study underpin the effectiveness of omalizumab for the treatment of CSU as well as CIndU and confirmed the role of serum total IgE before therapy as a predictive factor for treatment response, especially after 1 month. 

Since the phase 3 trial of omalizumab and subsequent approval for the treatment of CSU inadequately responsive to H1 receptor blockers, there have been a few reports of treatment effectiveness under real-world conditions. Nettis et al. [10] described the effect of omalizumab in 322 patients in a retrospective multicenter study. Consistent with our results, the study showed low total IgE and positive ASST as indicators of slow response (“slow responder”). Regarding ASST, Gericke et al. [11] had previously shown similar results in a cohort of 56 patients. The group of ASST-positive patients showed a prolonged time to response to omalizumab compared to patients with negative ASST [11]. Ghazanfar et al. [12] identified a negative histamine release test and a lack of concomitant angioedema as predictors of a rapid response in their retrospective study of 154 patients. No trends or significant differences were seen by the authors with respect to gender groups. A recent review concluded that for age, gender, and thyroid autoantibodies, there was strong evidence that these parameters had no association with omalizumab response [13]. The strongest evidence for a potential impact on response here was related to serum total IgE, as a large number of studies had shown that an elevated IgE at baseline was associated with a more rapid response to therapy compared with a low IgE [14, 15, 16, 17, 18]. These results are in agreement with ours. 

Surprisingly, with respect to response to omalizumab, we found a negative correlation with *Yersinia* IgA concentration, i.e., higher levels correlated with less improvement in CU-Q2oL at 6 months and UCT and CU-Q2oL at 12 months. Recently, an association of low IgA with low IgE in CSU was demonstrated and postulated [19]. We could not confirm this association for baseline values before initiation of therapy with omalizumab in our study. 

The present results confirm the generally very rapid response of CSU and CIndU to omalizumab already after the first dose, but also show that it is worthwhile to treat beyond 3 months because several patients benefit from this. Even over 2 years of treatment, effectiveness and improvement in quality of life are significantly improved and stable in CSU and CIndU (Figure 1). 

Our results showed female gender as a positive predictor of rapid response. In contrast, Fok et al. [13] found no association of gender with response to omalizumab in their review. 

In terms of limitations, the small number of CIndU patients should be mentioned. In addition, the significance of CSU is also limited in individual subgroup analyses with low frequencies. Overall, however, this study provides insight into the effectiveness in clinical practice. Since 2019, self-application of omalizumab has been approved from the fourth dose onwards, so that the systematic assessment of long-term efficacy with standardized instruments has been more difficult in everyday clinical practice since then, because the patients are no longer under such close medical supervision. 

In conclusion, the present study highlights the effectiveness of omalizumab for the treatment of chronic urticaria – CSU as well as CIndU. In particular, total IgE has been identified as a predictive marker for response in CSU. Real-life evidence, as presented in this study, is of great importance to evaluate the effect of a drug in real-world use. Especially with the emerging new systemic therapies for CSU, comparative analyses to other drugs in such observational studies become even more important. 

## Funding 

None. 

## Conflict of interest 

S. Traidl declares no conflict of interest. 

B. Wedi received fees for lectures or advisory boards of several hours from ALK-Abéllo, Bencard, Biocryst, CSL Behring, HAL Allergy, Novartis, Takeda, ThermoFisher Scientific. 

**Table 1.** Characteristics of patient cohorts.

	**Total (n = 144)**	**CSU (n = 112)**	**CIndU (n = 32)**	**p-value**
**Gender, n (%)**				0.239†
Female	85 (59.0)	69 (61.6)	16 (50.0)	
Male	59 (41.0)	43 (38.4)	16 (50.0)	
Age, years, mean ± SD	45.8 ± 15.9	46.7 ± 16.1	42.4 ± 15.0	0.157°
Age of onset, years, mean ± SD	37.6 ± 16.8	38.7 ± 16.9	33.9 ± 16.0	0.121°
**Smoking**, n (%)				0.070†
Yes	43 (29.9)	30 (26.8)	13 (40.6)	
No	88 (61.1)	69 (61.6)	19 (59.4)	
Not specified	13 (9.0)	13 (11.6)	–	
**Diseases**, n (%)				
Chronic spontaneous urticaria	127 (88.2)	112 (100.0)	15 (46.9)	
Angioedema	65 (45.1)	60 (53.6)	5 (15.6)	
Urticaria factitia	13 (9.0)	–	13 (40.6)	
Pressure urticaria	9 (6.3)	–	9 (28.1)	
Cold urticaria	2 (1.4)	–	2 (6.3)	
Solar urticaria	2 (1.4)	–	2 (6.3)	
Cholinergic urticaria	6 (4.2)	–	6 (18.8)	
**Months start of disease till Omalizumab (mean ± SD**)	45.7 ± 69.7	44.3 ± 74.3	50.6 ± 51.4	0.047°

^t^χ^2^-test; °Mann-Whitney U Test.

**Table 2.** Factors influencing the response to omalizumab in the group of patients with chronic spontaneous urticaria (CSU). Responder was defined as a UCT improvement of at least 3 points at 1 month.

	Responder		Non- Responder		p value	Odds ratio
	%	n	%	n		
**General**						
Total (n = 95)	66.3	63	33.7	32		
**Gender**						
Female (n = 58)	46.0	34	25.0	24	0.050	2.25 (1.00 – 6.56)
Male (n = 37)	66.3	29	75.0	8		
**Smoking**						
Yes (n = 24)	32.1	18	23.1	6	0.403	1.58 (0.54 – 4.61)
No (n = 58)	67.9	38	76.9	20		
**Concomitant diseases**						
**Helicobacter pylori**						
Yes (n = 19)	29.4	15	13.3	4	0.100	2.71 (0.81 – 9.11)
No (n = 62)	70.6	36	86.7	26		
**Thyroid disease**						
Yes (n = 23)	21.4	12	39.3	11	0.088	0.42 (0.16 – 1.14)
No (n = 61)	78.6	44	60.7	17		
**Erkrankung/Tests**						
**Angioedema**						
Yes (n = 53)	46.0	29	40.6	13	0.616	1.24 (0.52 – 2.95)
No (n = 42)	54.0	34	59.4	19		
**Autologous serum test**						
positive (n = 34)	35.3	18	61.5	10	0.031	0.34 (0.13 – 0.91)
negative (n = 43)	64.7	33	38.5	16		
**Laboratory examinations**						
**IgE**						
> 100 kU/l (n = 36)	58.1	28	71.4	8	0.059	2.54 (0.97 – 6.66)
≤ 100 kU/l (n = 49)	41.9	29	28.6	20		
> 50 kU/l (n = 53)	71.9	14	42.9	12	0.011	3.42 (1.33 – 8.80)
≤ 50 kU/l (n = 32)	28.1	16	57.1	16		
> 25 kU/l (n = 64)	89.5	51	46.4	13	0.001	9.81 (3.18 – 30.23)
≤ 25 kU/l (n = 21)	10.5	6	53.6	15		
**Yersinia serology**						
IgG positive (n = 37)	46.6	27	35.7	10	0.343	1.57 (0.62 – 3.97)
IgG negative (n = 49)	53.4	31	64.3	18		
IgA positive (n = 13)	15.8	9	14.3	4	0.856	1.13 (0.31 – 4.03)
IgA negative (n = 72)	84.2	48	85.7	24		
IgM positive (n = 27)	32.8	19	28.6	8	0.695	1.21 (0.45 – 3.27)
IgM negative (n = 59)	67.2	39	71.4	20		
**CRP**						
increased (n = 40)	47.5	28	40	12	0.504	1.34 (0.56 – 3.30)
Normal (n = 49)	52.5	31	60	18
